# Stabilizing Ultrathin CsPbBr_3_ Nanoplatelet Films for Deterministic Strong Light-Matter Coupling

**DOI:** 10.1021/acs.jpclett.5c03989

**Published:** 2026-02-25

**Authors:** Elizabeth O. Odewale, Isaac D. Boateng, Aaron S. Rury

**Affiliations:** Materials Structural Dynamics Laboratory, Department of Chemistry, 2954 Wayne State University, Detroit, Michigan 48202, United States

## Abstract

While the excitons in perovskite nanomaterials possess interesting properties central to their use in optoelectronic technologies, their instability limits real world applicability. In this study, we describe a method to stabilize the structures of cesium lead bromide (CsPbBr_3_) nanoplatelets (NPLs) in spin-casted films. We apply this method to design, fabricate, and characterize exciton cavity polaritons formed from these nanomaterials. These studies show that we can form exciton cavity polaritons from CsPbBr_3_ in precisely designed Fabry–Perot microresonators and suggest that the structures of these materials can be stabilized toward their use in other solution-processed optoelectronic devices.

## Graphical abstract

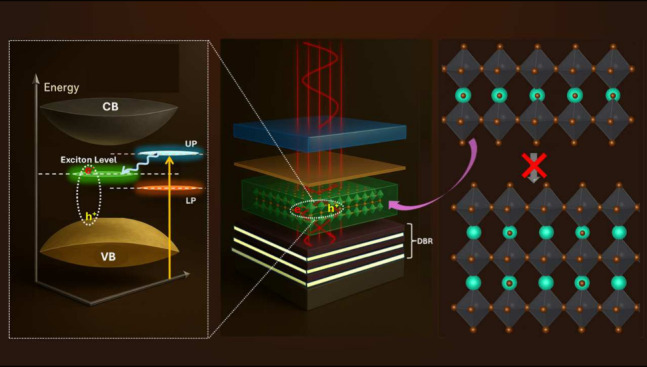

The formation of exciton cavity polariton states through strong light-matter coupling enables researchers to leverage the long-range spatial coherence of photons with the nonlinear behavior of electronic excitations in materials.^1–5^ Many material platforms have been leveraged to form these hybrid light-matter states including molecular chromophores,^6–13^ biological systems,^14^ precision-fabricated quantum wells,^15–17^ and solution-processed nanomaterials such as quantum dots^18,19^ and nanoplatelets.^20–23^ The underlying properties of the material used to form cavity polaritons can enable the observation of novel physical and chemical phenomena not observed outside of the electromagnetic cavity including Bose–Einstein condensation^24^ and amended photochemistry.^25^

Metal halide perovskites (MHPs) are a class of ionic, crystalline materials composed of octahedra centered typically with main group metal cations surrounded by halide anions. When formed into cubic crystal lattices, these materials take the general chemical formula ABX_3_ where the A-sites are occupied by 1+ cations and the B and X are the metal and halide ion, respectively.^26^ The A-site cations act to balance the negative charge of the BX_3_ structure. When large enough A-site cations are employed, one can drive formation of layered, perovskite-like materials whose structures cause strong quantum and dielectric confinement of electronic excitations within self-assembled quantum wells, which increases exciton binding energies (E_B_) significantly.^27–31^ Large E_B_ values in layered MHP materials also increase the oscillator strengths associated with their optical transitions, which helps these systems reach cavity-enhanced strong light-matter coupling conditions at room temperature when formed into these self-assembled quantum wells.

The formation of exciton cavity polaritons using layered, MHP-like materials has provided experimental samples where researchers can investigate exotic physical phenomena.^32,33^ These phenomena including interactions between Bose–Einstein condensates (BECs) formed from polarization-coupled cavity modes^34^ and unidirectional BEC propagation due to topological protection.^35^ Additionally, the properties of excitons in MHP materials have enabled researchers to apply cavity polaritons they form in simulating the behavior of complex quantum spin interactions when patterned into interacting BEC lattices. Despite these important findings, cavity polariton samples formed from excitons in MHP-like materials suffer from specific limitations.^36,37^

Many studies demonstrating strong light-matter coupling using excitons from MHP-like materials are formed through complex fabrication steps that can necessitate mechanical exfoliation^36,37^ or crystal growth within a microresonator.^35^ These methods cannot be controlled precisely and lead to cavity polariton formation in multimode electromagnetic resonators, which causes complexities in spectroscopic characterization.^38^ These aspects of sample fabrication limit the scalable use of cavity polariton devices formed from layered MHP-like materials. Confining the layered MHP materials into the nanometer-scale lateral dimensions results in the formation of so-called quasi-2D nanoplatelets (NPLs), which can be dispersed in solutions that are deposited into electromagnetic cavities deterministically. These nanomaterials differ from the bulk self-assembled quantum wells in at least three important ways. First, the increased quantum confinement causes a blue shift in excitonic energy and allows for tunable exciton energies by controlling the thickness down to the monolayer level. Second, the nanoplatelets’ excitons possess larger oscillator strength per unit area, which drives stronger interactions with external electromagnetic fields. Third, the spectral line width in the nanoplatelets is narrower compared to the bulk layered MHP due to the presence of grain boundaries and defects in the latter.^39–41^ These features make the MHP nanoplatelets promising for fundamental studies of exciton cavity polaritons. However, MHP NPLs are inherently less stable than covalent semiconductors such as the III–V group systems.^22,42–47^ This effect results from the tendency of NPLs to aggregate into thicker structures.

For example, researchers have demonstrated that cesium lead bromide (CsPbBr_3_) NPLs will tend to form thicker and thicker layers when cast into thin films from liquid drops through both heat and light exposure.^48–51^ Figure 1(a) shows the temporal evolution of the NPL structure and the spectra schematically. Starting from a distribution of n = 2 CsPbBr_3_ nanoplatelets at time t_0_, the individual nanoplatelets can react to form some n = 3 species at t_1_. Those thicker nanomaterials then mix into the distribution of n = 2 species. Letting the sample sit longer to time t_2_ results in further chemical conversion to the n = 4 species among some of the smaller nanoplatelets. Ultimately, the nanoplatelets in the sample combine to form the n = 5 species, which has a structure like that of cubic CsPbBr_3_ nanocubes, as shown in Figure 1 after the time t_3_. As the atomic structures of these nanomaterials evolve temporally, so do their electronic states. Figure 1 shows that the bandgap (E_*g*_) and exciton binding energy (E_*B*_) both reduce as the individual particles become larger. These trends result from the reduced quantum and dielectric confinement of the electrons and holes induced by increasing the particle lattice widths. These changes to the electronic structures of the distinct nanomaterial species can distinguish their associated absorption spectra.

**Figure 1:**
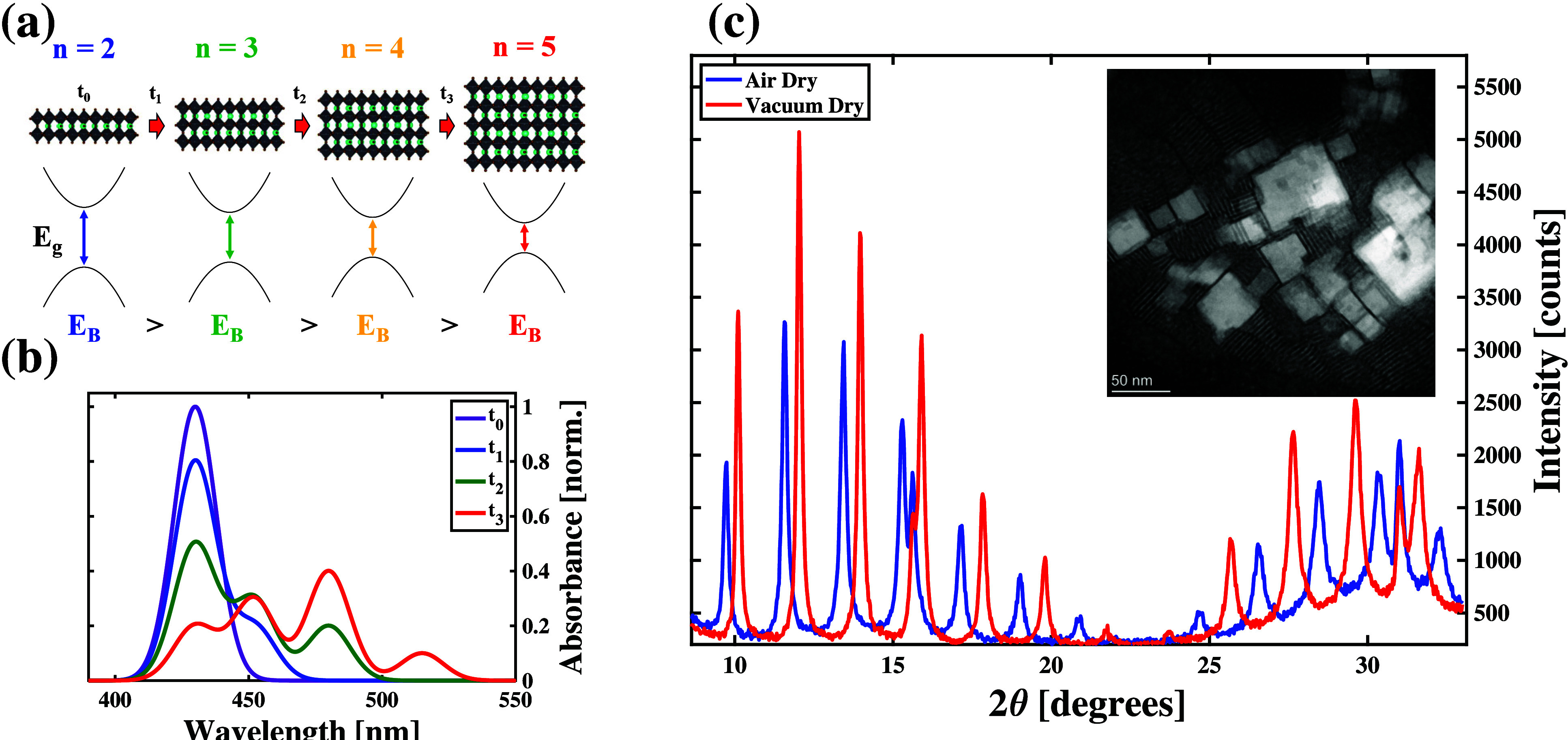
(a) Schematic representation of the structural evolution of CsPbBr_3_ nanoplatelets (NPLs) when exposed to ambient conditions without applying stabilization methods. As time progresses, the growth of these particles along the confinement direction results in a stepwise reduction of both the material’s bandgap (E_g_) and the exciton binding energy (E_B_). (b) Model excitonic light absorption spectra consistent with the structural changes shown in the left panel. These changes with structural evolution indicate that stabilization methods are necessary to sustain absorption at specific wavelength, which is crucial for exciton cavity polariton formation. (c) Comparison between the X-ray diffraction patterns of *n* = 2 CsPbBr_3_ NPL thin films measured before (before) and after (red) applying the processing steps described in the main text. Inset: representative transmission electron micrograph of *n* = 2 CsPbBr_3_ NPLs drop cast onto a copper grid.

Figure 1(b) shows the evolution of the excitonic contribution to the absorption spectrum of the sample, as the structures of its constituent nanomaterials change with time. Initially, the excitonic peak from the n = 2 nanoplatelet species is the only feature present in the absorption spectrum. As time progresses and larger nanoplatelet structures form, the excitonic peaks associated with those structures appear in the absorption spectrum with contributions consistent with their oscillator strengths and sample concentrations. For example, at t_1_ the absorption spectrum shows contributions from excitons in both the n = 2 and n = 3 species. At t_2_, the feature associated with the n = 4 species’ excitons appears in the absorption spectrum, while we find the n = 5 exciton contributes enough to appear in the modeled spectrum. Since cavity polariton formation depends on careful design of microresonators fabricated in sequential deposition of different material layers, the instability of a light absorbing material would be detrimental to leveraging hybrid light-matter states for the applications noted above. The successful application of these solution-processed nanomaterials to cavity polaritonics will rely on methods to stabilize their structures in thin film morphologies during the fabrication of deterministically design cavity architectures.

In this study, we develop a strategy to stabilize the structures of ultrathin, n = 2 CsPbBr_3_ NPLs when cast into neat thin films using spin processing methods. We then determine the refractive index of these films to design microcavities capable of reaching the strong light-matter coupling limit. By fabricating and characterizing these samples experimentally, we found results consistent with cavity polariton formation. Our results provide insights into the stabilization of nanomaterial thin films, characterization of their anisotropic optical properties, and methods to form hybrid light-matter states toward exploiting exciting materials properties, in polariton behavior using more reproducible and scalable methods.

We synthesize the NPLs in solution according to methods established in past reports.^40^ The inset of Figure 1(c) shows a representative transmission electron micrograph of the NPLs we form. We focus our study on thin films of n = 2 CsPbBr_3_ NPLs cast from suspensions in hexane by using a spin processor. Previous studies show that NPLs in drop-cast films formed from similar suspensions lie parallel to the substrate surface, which we call the face-down orientation.^52,53^ Periodic peaks in X-ray diffraction (XRD) patterns we measure on these films result from Bragg reflections due to formation of interparticle superlattices and confirm the facedown orientation of the NPLs in our samples, as shown in Figure 1(c). The facedown orientation of the NPLs enables us to couple to the bright, in-plane excitons of these nanoparticles for spectroscopic characterization and exciton cavity polariton formation.^52,53^ Additionally, we find minor peaks at 2θ values of 15.61° and 31.01°, which are reported in the literature as belonging to the edge-up orientation of CsPbBr_3_ NPLs.^52,53^ The NPLs in an edge-up orientation arrange themselves such that their long axes lie perpendicular to the substrate surface. Despite their low intensities, the presence of these peaks suggests that a small fraction of nanoplatelets in our as-prepared samples adopt this orientation.

Our spectroscopic data confirm the salient features of the simple model considered in panels Figure 1(a) and Figure 1(b). As seen in Figure 2(a), the absorption spectra of a 100 nm thick film of CsPbBr_3_ n = 2 nanoplatelets degrades significantly over the first 4 days following its initial fabrication, as indicated by the growth of the absorption peak of the exciton assigned to the exciton in n = 3 nanoplatelets in previous studies.^48^ As noted in the Supporting Information (SI), we did not undertake any postprocessing of this thin film following its fabrication. We then determined that sequential heating, drying, cooling, and exposure to vacuum conditions stabilize the microscopic atomic structures of the n = 2 nanoplatelets. As shown in Figure 2(b), the absorption spectrum of the sample subjected to those postprocessing steps does not change in appreciable ways over the course of 10 days of storage under ambient conditions. The lack of any significant change in the absorption spectra of samples processed with these steps following their initial fabrication indicates that the underlying microscopic structures of the CsPbBr_3_ nanoplatelets do not evolve after we complete them. The XRD patterns reflected by our samples before and after applying these processing steps help explain the microscopic processes taking place that stabilize the structures of the film’s underlying NPLs.

**Figure 2:**
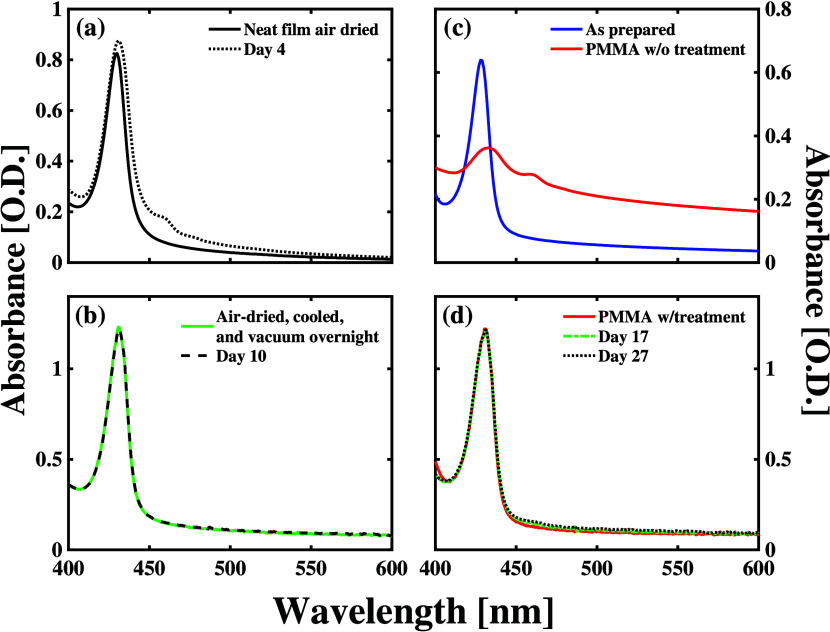
(a) Comparison between the absorption spectrum of a CsPbBr_3_ NPL film taken immediately after its fabrication (solid line) to that of the same film after storing it under ambient conditions for 4 days (dotted line). (b) Comparison between the absorption spectrum of a CsPbBr_3_ NPL film taken after subjecting the material to air drying, refrigeration, and storing under vacuum for 12 h (solid line) to that of the same film after leaving it under ambient conditions for 10 days (dashed line). (c) Comparison between the absorption spectrum of a CsPbBr_3_ NPL film taken immediately after it fabrication (blue line) to that of the same film after depositing a layer of poly­(methyl methacrylate) (PMMA) thereafter (red line). (d) Comparison between the absorption spectrum of a CsPbBr_3_ NPL film taken after subjecting the material to air drying, refrigeration, and storing under vacuum for 12 h and coating it with 150 nm PMMA layer (solid line) to that of the same film after storing it under ambient conditions for 17 days (dash-dotted line) and 27 days (dotted line).

Figure 1(c) compares the XRD patterns we observe prior to and after subjecting our CsPbBr_3_ NPL thin films to the processing steps describe above. As seen by this comparison, we find that the structure of the underlying film changes due to the processing steps. These changes manifest themselves as an increase in the XRD peak intensities, shifts in the positions of the XRD modulation peaks, widening of the spacing between the Bragg modulation peaks, and a reduction in the contribution of the edge-up orientation to the overall pattern. The increased peak heights suggest that application of the postprocessing steps improves the overall crystallinity of thin films. The shift of the Bragg peaks that modulate the XRD pattern indicate that we stabilize each CsPbBr_3_ NPL such that the Pb–Pb distances reduce coincidently across the particles that comprise the thin film, as established through structural studies of similar materials formed with differently sized A-site cations.^53^ The spacing between the Bragg peaks increases by 0.05°, which indicates that the interparticle separation within the superlattice structure reduces. Based on these changes observed in XRD measurements, we propose that the postprocessing methods we employed help stabilize the sublattice of organic ligands that cap the individual NPLs during the solution-phase synthesis. These changes indicate improved crystallinity and stabilization of the organic sublattice, which allows tighter packing of the CsPbBr_3_ NPLs within the superlattice. Notably, the intensity of the minor peaks generally ascribed to edge-up oriented NPLs decreases, indicating that the postprocessing further enhances the homogeneity of the film. This more uniform, face-down alignment facilitates stronger coupling to cavity photons in our spectroscopic measurements. Overall, the observed XRD changes confirm that the postprocessing steps help align, structurally relax, and stabilize the NPLs in their dominant face-down orientation, thereby facilitating strong light–matter coupling.

To further stabilize these light absorbing thin films toward their use in forming cavity polaritons, we tested the deposition of poly­(methyl methacrylate) (PMMA) layers over the n = 2 CsPbBr_3_ nanoplatelet thin film. As shown in Figure 2(c), depositing and curing a PMMA layer on the CsPbBr_3_ nanoplatelet film immediately following its fabrication degrades the n = 2 excitonic peak significantly and causes the appearance of a peak near the wavelength assigned to the exciton in n = 3 nanoplatelets. However, when we concentrate the nanoplatelets in a hexane dispersion and follow the steps shown to stabilize the neat thin films described above, we can maintain the characteristic absorption spectrum of the n = 2 CsPbBr_3_ nanoplatelets after depositing and curing the PMMA layer, as seen in Figure 2(d). Ellipsometric measurements on capped silicon substrates indicate that we form a ∼ 250 nm layer of PMMA using the processing steps described in SI. The absorption spectrum can be stabilized following capping with PMMA for at least 27 days when using these steps, as shown in Figure 2(d). These results indicate that our sample postprocessing methods can be applied to stabilize the microscopic structures of the CsPbBr_3_ NPL thin films adequately toward their use in cavity polariton formation. As shown in , neither drying under ambient conditions nor refrigerating the CsPbBr_3_ nanoplatelet films overnight stabilizes the light absorbing layers enough to reduce their degradation following deposition and curing the PMMA layer.

It is worth emphasizing that the primary improvement in stability arises from careful postsynthetic processing rather than from encapsulation alone. Steps such as air drying, refrigeration, and vacuum drying allow for subtle structural rearrangements within the perovskite lattice and enhance the optical density of the films, leading to improved stability of the ligand-passivated perovskite moieties. Following this pretreatment, PMMA encapsulation provides an additional layer of protection, acting as a barrier against ambient moisture and oxygen. While the polymer layer alone cannot fully compensate for inadequate preprocessing, it helps preserve the structural and optical improvements achieved through postsynthetic treatment, enabling reliable integration of the nanoplatelets into microcavity devices.

Other polymers could provide similar stabilization effects if they are processed carefully. For the CsPbBr_3_ nanoplatelets in particular, the solvent used to dissolve the polymer must be compatible with the nanoplatelet film, meaning it should not dissolve or otherwise damage the perovskite layer formed from its original dispersion solvent. Optimizing the deposition method and thermal treatment is also important to avoid film degradation. Overall, the combination of postsynthetic processing to stabilize the perovskite moieties, followed by polymer encapsulation for film protection, represents a practical and versatile method to maintain the properties of CsPbBr_3_ nanoplatelets for optoelectronic and polaritonic applications.

Figure 3 illustrates the mechanism of strong light–matter coupling in a Fabry–Perot microcavity containing CsPbBr_3_ nanoplatelets. On the left, 2-monolayer CsPbBr_3_ nanoplatelets are sandwiched between two mirrors, with the exciton depicted as an electron–hole pair. The overlaid sinusoidal wave represents the standing cavity electric field, showing the likelihood of interaction between the exciton and the cavity photon. The right side of the schematic shows a spatial view of the cavity layers, highlighting the alternating high- and low-index regions of the distributed Bragg reflector, the active nanoplatelet layer, the polymer layer, and the aluminum mirror representing a typical microcavity used in the study. We modeled the electric field propagation through the structure and combined the wave with a refractive index plot to illustrate how the electromagnetic wave travels across the different layers. The combination of the spatial schematic and the field simulation demonstrates how the confined optical field can overlap with excitonic transition to enable coherent energy exchange that drives strong light-matter interaction.

**Figure 3:**
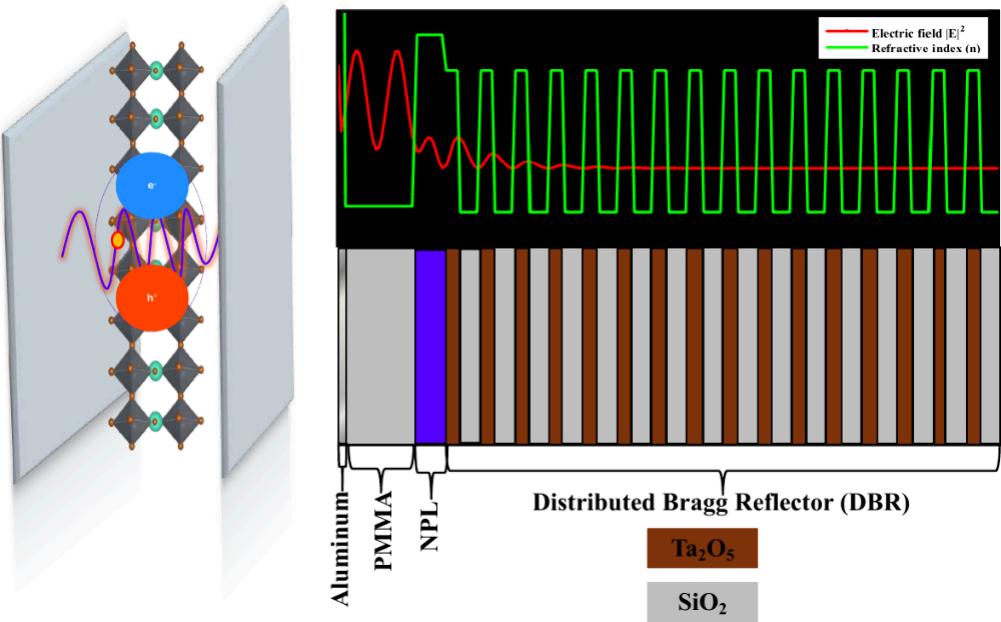
Left panel: schematic of a 2-monolayer CsPbBr_3_ nanoplatelet (NPL) embedded between cavity mirrors, showing an exciton (electron–hole pair) interacting with the oscillating cavity electric field. Top right panel: simulated electric-field intensity (red) and refractive index profile (green) across a microcavity designed to couple cavity photons to excitons in CsPbBr_3_ that were fabricated in this study. Bottom right panel: spatial representation of the cavity layers, including the DBR (alternating Ta_2_O_5_ and SiO_2_), NPL layer (bluish purple), PMMA spacer, and aluminum mirror. The simulation highlights light confinement in the cavity and its overlap with the NPL layer to induce strong light-matter coupling.

As seen by Figure 3, the formation of cavity polaritons that possess deterministic properties necessitates capping our CsPbBr_3_ film structures with an additional mirror. Building off our previous studies of cavity polaritons formed from metalloporphyrin chromophores,^10–13^ we capped our CsPbBr_3_ film structures with ∼15 nm aluminum layers to achieve at least 90% reflectivity. We formed control samples to assess the stability of absorption spectra characterizing the CsPbBr_3_ nanoplatelet films within our microcavity samples. As shown in Figure S3, we maintain a prominent absorption peak consistent with the n = 2 CsPbBr_3_ nanoplatelet exciton following our capping of the PMMA with Al. We come to this conclusion by noting that the light transmission through both samples is reduced by 70% at the excitonic peak relative to the background. Additionally, the salient features of the samples’ reflection spectra remain unchanged after depositing the metal film onto the PMMA layer. The stability of the sample through these processing steps indicates its suitability for cavity polariton formation. We used transfer matrix methods based on pertinent material optical parameters to design our cavity to ensure coupling between the photons confined by the reflecting mirrors and the n = 2 excitons of the CsPbBr_3_ excitons, as explained in the SI.

The panels Figure 4(a) and Figure 4(b) show the angle-resolved reflection spectra of our cavity and control samples that we find from experiment and a computational model, respectively. As seen in Figure 4(a), the discernible peaks in our experimental spectra are split in energy and shift to shorter wavelengths (higher energies) as we increase the angle of incidence, θ_*inc*_, which is the telltale sign of exciton cavity polariton formation. The higher frequency of two peaks we observe at 415 nm for θ_*inc*_ = 10° shifts more significantly than the frequency we observe at 440 nm at the same incidence angle as would be expected of the upper polariton (UP) state since it contains more photonic content than the lower polariton (LP) for blue-detuned conditions. Figure 4(b) shows that our transfer matrix calculations reproduce the salient features of the experimental spectra when we use material properties we established via Kramers–Kronig and cavity parameters detailed in the Methods section found in the SI. Comparing the insets in Figure 4(a) and Figure 4(b), we see that the TM calculations produce a spectrum for θ_*inc*_ = 10° that matches the experimental results qualitatively and possesses only an offset in the baseline reflection, which likely results from the imperfect collection of the light field reflected from the sample in our experiments. The qualitative agreement of our experimental and computational results indicates that we produced cavity polaritons from our stabilized CsPbBr_3_ NPLs.

**Figure 4:**
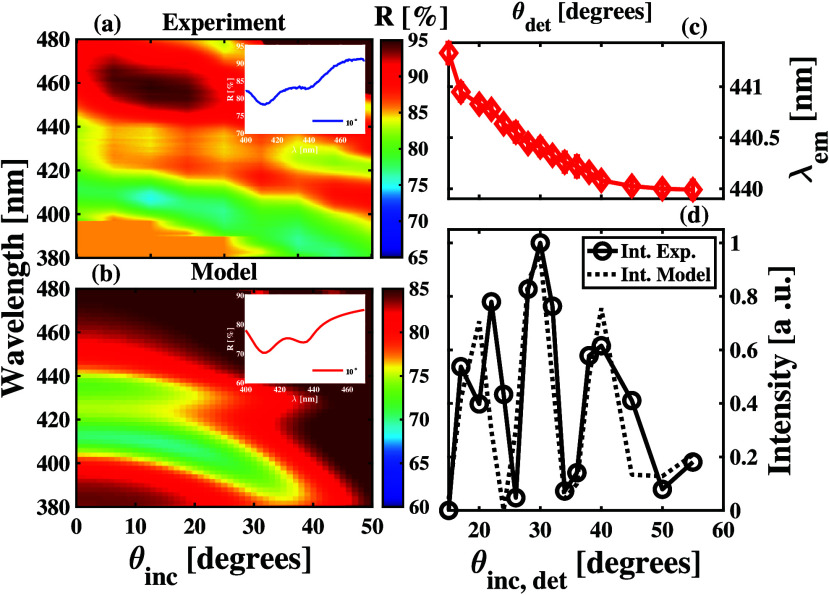
Angle-resolved reflection spectrum of a Fabry–Perot cavity loaded with CsPbBr_3_ nanoplatelets measured experimentally (a) and modeled computationally (b). Insets show the reflection spectra found at θ_*inc*_ = 10°. Detection angle-resolved energy of a 441 nm peak in the photoluminescence (PL) spectrum of a Fabry–Perot cavity loaded with CsPbBr_3_ nanoplatelets (c). 95% confidence intervals in estimates of the peak positions are shown as errorbars on top of diamonds for each θ_det_ point where a PL measurement was made. Angle-dependent intensity of the dispersive PL peak measured at 400 nm (d) measured experimentally (circles, solid line) compared to the results of model described in the text (dotted line).

Angle-resolved photoluminescence measurements have been used extensively to assess exciton cavity polariton formation and properties in semiconductor nanostructures.^16^ Figure 4(c) shows the angular dependence of the PL peak we observe at 441 nm at θ_*inc*_ = 10° after exciting a cavity sample with a 3.06 eV CW laser, as detailed in the SI. As we increase θ_*inc*_, the angle of detecting the PL signal (θ_det_) changes by the same amount and we find that the peak position shifts to shorter wavelengths (higher energies), which is not observed for control sample that does not contain a DBR mirror (Figure S5). Additionally, we find that the peak position remains at 440 nm as we increase and surpass θ_det_ equal to 40°. This behavior is expected from the LP state of the strongly coupled light-matter system.

Additionally, Figure 4(d) shows that the intensity of this peak oscillates as we rotate the sample relative to the excitation laser source. Since this rotation changes the angle of both the incidence (θ_*inc*_) and detection (θ_det_) in our measurements, we model the intensity using the equation

$$I\left(\theta_{inc},\theta_{\det}\right)=I_{0}e^{-{\left[\left(\theta_{inc}-\theta_{0}\right)/2\Delta \theta \right]}^{2}}\;\cos^{2}\left[\pi \frac{d_{eff}\sin\left(\theta_{\det}\right)}{\lambda_{em}}\right]$$

^54,55^ The first term in this model accounts for the change in the resonance condition between the incident laser photons and the UP state of the sample, as we considered previously.^56^ The second term of this model accounts for the expected interference present in light emission from Fabry–Perot cavities, which depends on the effective length of the cavity *d*_*eff*_ and the wavelength of the emitted photons, λ_*em*_.^54^

Comparison between our experimental and model results shown in Figure 4(d) demonstrates that parameter values of θ_0_ = 30°, Δθ = 10°, and *d*_*eff*_/λ_*em*_ = 6.12 enable us to explain the intensity oscillations sufficiently. Nonlinear regression analysis of the angle-dependent intensity estimates similar parameter values, as detailed in the SI and shown in Figure S6. These results further support our conclusion that we form exciton cavity polaritons from the NPLs we embedded in our Fabry–Perot structures. The light emission peak must arise from an exciton-like state while the dispersion of the peak energy and oscillations of the peak intensity must arise from a photon-like state. These features can only arise from the same peak at the same time if we have formed hybrid light-matter states. The design and characterization of our cavity samples suggest steps one can take to further optimize these structures for strong light-matter coupling.

The polymer layer influences the microcavity primarily through its refractive index, while the polymer thickness is adjusted to satisfy the cavity resonance condition. Increasing the polymer refractive index modifies the cavity photon dispersion,^57,58^ leading to flatter polariton branches as predicted by the inverse dependence of the energy of the cavity photon on the effective refractive index.^59^ Our simulations show this flattening to be particularly prominent in the lower polariton branch, accompanied by a reduction in intensity and a small red-shift of the peak energy at normal incidence. Furthermore, a higher refractive index reduces the refractive index mismatch at the polymer-nanoplatelet interface, thereby decreasing the Fresnel reflection and slightly altering the cavity field distribution within the nanoplatelet layer. These effects may occur without a significant change in the Rabi splitting if the exciton density determined by the thickness of the absorber remains the same.

Despite how readily the nanocubes form and their perceived stability compared to the nanoplatelets, the nanoplatelets offer distinct advantages as the active layer for polariton formation in microcavities.^60^ Compared to nanocubes, the platelets combine large excitonic oscillator strength, narrow excitonic line widths, tunable bandgap, and planar dipole orientation, making them particularly well suited for exploring strong light–matter coupling in a Fabry–Perot microcavity.^41,61–63^ One key advantage is the ability to tune the excitonic bandgap by controlling the number of monolayers, allowing precise alignment with the cavity photon without altering the chemical composition of the perovskite lattice.

In this study, the thickness of CsPbBr_3_ nanoplatelets was carefully controlled during synthesis to achieve the desired monolayer number, enabling fine-tuning of the exciton energy across the visible spectrum. By contrast, nanocube bandgaps are primarily tuned via chemical substitution,^53,64^ providing less continuous control and altering the lattice structure. Also, the quasi-2D geometry of nanoplatelets leads to stronger quantum confinement and higher exciton binding energies than nanocubes, stabilizing the nanoplatelets’ excitons and enhancing their interaction with cavity photons. The nanoplatelets also exhibit longer exciton coherence lengths and narrower homogeneous line widths,^65–67^ which improve the visibility of polariton peaks.

In addition, the planar morphology and anisotropic shape allow preferential in-plane orientation during film deposition, maximizing overlap with the cavity electric field, whereas nanocubes tend to adopt random orientations that reduce effective coupling.^68^ Together with the higher effective density of aligned dipoles in the film, these features increase the collective light–matter interaction strength and Rabi splitting. Studies of polariton formation in other colloidal nanoplatelet systems, such as CdSe, further confirm the efficiency of nanoplatelet exciton states in hybridizing with cavity photons.^20,69,70^ Overall, these structural and optical properties make CsPbBr_3_ nanoplatelets especially advantageous for microcavity polariton studies and the development of tunable strong-coupling devices, despite the difficulty around stabilizing and incorporating them into microcavities.

Our results suggest that MHP NPLs may be more widely applicable with appropriate stabilization. While we have focused on the formation of exciton cavity polaritons in this study, there is reason to believe that the stabilization methods described in this study will be useful to fabricate a wide array of optoelectronic devices including lasers, LEDS, and photosensors. Additionally, one could use the methods detailed in this study to form other electronic devices including transistors from MHP NPLs, which could further enable one’s ability to process integrated circuits using solution chemistry. Further studies are needed to determine what additional steps are needed beyond those described here to stabilize the structures of these fascinating materials toward their application in those devices, which are beyond our consideration here.

In conclusion, we have demonstrated a method to stabilize the structures of CsPbBr_3_ nanoplatelets toward their use in cavity polaritons formed from the exciton transitions in these metal halide perovskite (MHP)-like materials. By annealing films prepared via spin-processing methods through several steps and capping them with a polymer layer, we observe a maintenance of the main excitonic peak characteristic of n = 2 CsPbBr_3_ nanoplatelets for 10 times longer than measured in untreated films. By modeling the optical properties of these films using transfer matrix methods, we are able to design Fabry–Perot microresonators capable of coupling to the n = 2 CsPbBr_3_ nanoplatelet exciton transitions of these films. Comparisons between experimental and computational reflection spectra of the designed cavity both possess the characteristic features of polaritonic spectra, which indicates our ability to reach the strong light-matter coupling limit using our methods. We leveraged angle-resolved photoluminescence spectroscopy to support this conclusion. These results indicate that MHP-like materials can be stabilized sufficiently to form exciton cavity polaritons in more precise and reproducible ways than reported previously for their use in optoelectronics devices and tests of fundamental quantum phenomena.

## Supplementary Material





## Notes

Experimental and computational methods, absorption spectra of NPL films formed using different processing methods, transmission and reflection spectra of Al-capped polymer-NPL films, comparisons between the angle-dependence of PL peaks in control and cavity samples, and comparisons between experimental PL intensity to nonlinear regression fits to an angle-dependent model (PDF)
Transparent Peer Review report available (PDF)
