# Supplementary material for: Stabilizing Ultrathin CsPbBr3 Nanoplatelet Films for Deterministic Strong Light-Matter Coupling
Source: J Phys Chem Lett. 2026 Feb 25;17(10):2877–85. doi: 10.1021/acs.jpclett.5c03989 (PMC12990107; doi:10.1021/acs.jpclett.5c03989)
Supplement: Supplementary file 1 [file jz5c03989_si_001.pdf]

Supporting Information for Stabilizing Ultrathin $CsPbBr_3$ Nanoplatelet Films for Deterministic Strong Light-Matter Coupling

Elizabeth O. Odewale, Isaac D. Boateng, and Aaron S. Rury*

Materials Structural Dynamics Laboratory, Department of Chemistry, Wayne State University, Detroit, MI, USA 48202

E-mail: arury@wayne.edu

Contents

List of Figures **S3**

S1 Methods **S6**

S1.1 Experimental Methods S6

S1.1.1 Materials S6

S1.1.2 Synthesis of n = 2 Cesium Lead Bromide Nanoplatelets S6

S1.1.3 Structural Characterization S6

S1.1.4 Purification and Film Deposition S7

S1.1.5 PMMA Coating and Thermal Annealing S7

S1.1.6 Design, Fabrication, and Characterization of Polaritons and Control Samples S8

S1.1.7 Optical Characterization S8

S1.2 Computational Methods S9

S2 Comparison of Absorption of $CsPbBr_3$ Nanoplatelet Thin Films Formed under Different Processing Conditions **S9**

S3 Effects of Capping Stable $CsPbBr_3$ Films with Aluminum **S11**

S4 Structure of Fabry-Pérot Micro-resonators for Exciton Cavity Polariton Formation **S12**

S5 Comparison of Angle-Resolved Photoluminescence Peaks between Control and Cavity Samples **S12**

S6 Comparison of Angle-Resolved Photoluminescence Intensity from Experiment and Model **S13**

References **S15**

List of Figures

S1 **Top left panel**: comparison between a thin film comprised of n = 2 $CsPbBr_3$ NPLs immediately following is fabrication to that of the same sample 4 days later, which shows an relatively increased baseline and a peak corresponding to the n = 3 $CsPbBr_3$ NPL species. **Top right panel**: comparison between a thin film comprised of n = 2 $CsPbBr_3$ NPLs following 5 minutes of heating at 70° after initial fabrication to that of the same sample 4 days later, which shows an relatively increased baseline and a peak corresponding to the n = 3 $CsPbBr_3$ NPL species. **Bottom left panel**: comparison between a thin film comprised of n = 2 $CsPbBr_3$ NPLs following 5 minutes of heating at 100° after initial fabrication to that of the same sample 4 days later, which shows an relatively increased baseline and a peak corresponding to the n = 3 $CsPbBr_3$ NPL species. **Bottom right panel**: comparison between a thin film comprised of n = 2 $CsPbBr_3$ NPLs following air drying, refrigeration, and vacuum drying for 12 hours after initial fabrication to that of the same sample 10 days later, which shows no signs of a peak corresponding to the n = 3 $CsPbBr_3$ NPL species. S10

S2 **Top left panel**: comparison between a thin film comprised of n = 2 $CsPbBr_3$ NPLs immediately following is fabrication to that of the same sample after encapsulation with a ~250 nm PMMA layer, which shows an relatively increased baseline and a peak corresponding to the n = 3 $CsPbBr_3$ NPL species. **Top right panel**: comparison between a thin film comprised of n = 2 $CsPbBr_3$ NPLs immediately after initial fabrication to that of an air-dried sample and a refrigerated sample after encapsulation with ~250 nm PMMA layers, which both show relatively increased baselines and shifts of the main excitonic absorption peak. **Bottom left panel**: comparison between a thin film comprised of n = 2 $CsPbBr_3$ NPLs following air drying, refrigeration, and vacuum drying for 12 hours after initial fabrication to that of the same sample after encapsulation with a ~250 nm PMMA layer, which shows no signs of a peak corresponding to the n = 3 $CsPbBr_3$ NPL species. **Bottom right panel**: time evolution of the absorption spectrum of the sample shown in the bottom left panel at 1 day, 17 days, and 27 days after its initial fabrication, which shows no signs of additional peaks. S11

S3 **Left panels**: transmission (top) and reflection (bottom) spectra of a stabilized n = 2 n = 2 $CsPbBr_3$ NPL thin film after being encapsulated with a ~250 nm PMMA layer. **Right panels**: transmission (top) and reflection (bottom) spectra of a PMMA-capped, stabilized n = 2 n = 2 $CsPbBr_3$ NPL thin film after being encapsulated with a ~15 nm Al layer. S12

S4 Schematic comparison between the structures Fabry-Pérot (left) and control (right) samples we used to assess the effects of strong light-matter coupling with $CsPbBr_3$ NPL films. Al mirror is a ~15 nm layer of aluminum, PMMA layer is a 200-300 nm film of polymethyl methyacrylate, NPL layer is the ~100 nm thin film of $CsPbBr_3$ nanoplatelets, DBR is a distributed Bragg reflection, and each sample is formed on an optical quality silicon dioxide (SiO_2) substrate. S13

S5 Comparison of the detection angle (θ_{det}) dependence of the 441 nm peak in the photoluminescence spectrum of a $CsPbBr_3$ NPL film embedded in a precision fabricated Fabry-Pérot micro-resonator (red squares) to that of a 438 nm peak in the control sample shown in Figure 4 (blue circles). S14

S6 Comparison of the dependence of the 441 nm peak in the photoluminescence spectrum of a $CsPbBr_3$ NPL film embedded in a precision fabricated Fabry-Pérot micro-resonator on incidence (θ_{inc}) and detection (θ_{det})angle (θ_{det}) dependence (black circles) to that of a nonlinear regression fit to Eqn. (S1) (dotted line. S15

S1 Methods

S1.1 Experimental Methods

S1.1.1 Materials

Octadecene (ODE, 98%), oleic acid (OA, technical grade, 90%), oleylamine (OLAM, technical grade, 70%), hydrobromic acid (HBr, 48% in water), lead(II) bromide ($PbBr_2$, 99.999%), and cesium carbonate (Cs_2CO_3, 99.9%) were purchased from Sigma-Aldrich and used as received unless otherwise stated. Dimethylformamide (DMF, anhydrous), acetone, isopropanol, and hexane were obtained from Fischer Chemical. Polymethyl methacrylate (PMMA, 6% in anisole, A6) was purchased from Kayaku Advanced Materials, Inc. Cesium oleate precursor was prepared following established procedures. Silicon and fused silica wafers MTI Corporation were used as substrates for film deposition.

S1.1.2 Synthesis of n = 2 Cesium Lead Bromide Nanoplatelets

Nanoplatelets were synthesized under ambient conditions. In a 20 mL glass vial, 1.25 mL ODE, 0.125 mL OA, and 0.125 mL OLAM were combined and mixed at room temperature. To this mixture, 20 μL of HBr was added to initiate the formation of thin nanoplatelets. The volume of HBr was chosen based on its known influence on nanoplatelet thickness. Subsequently, 0.2 mL of Cs-oleate, preheated to approximately 90 °C to ensure complete dissolution, was added. Following this, 0.3 mL of a 0.4 M $PbBr_2$ solution in DMF was rapidly injected into the reaction mixture. Turbidity developed within seconds, indicating nanoplatelet formation. After 10 seconds, the reaction was quenched by adding 5 mL of acetone, which resulted in a visible yellowish-green color typical of n = 2 colloidal CsPbBr3 nanoplatelets.

S1.1.3 Structural Characterization

Transmission Electron Microscope images were collected using Talos F200X in HAADF-STEM mode at 200 kV. EDS acquisition for composition analysis was performed using the same instru-

ment in the TEM mode. For all imaging, the nanoplatelets colloid in hexane was dropcasted on a carbon-coated copper TEM grid and loaded onto a single-tilt sample holder. X-ray diffraction (XRD) measurements were performed with a Bruker D2 Phaser operating at 30 KV and 10 mA using copper radiation and a LYNXEYE™ detector. Samples for the XRD measurements were prepared by spin coating the nanoplatelets on fragments of silicon wafer, which was then mounted on a zero-diffraction silicon sample holder. All XRD measurements were carried out at 300 K

S1.1.4 Purification and Film Deposition

The reaction mixture was transferred to a 15 mL centrifuge tube and centrifuged at 4000 rpm for 7 minutes. The supernatant was discarded, and the precipitate was redispersed in hexane, followed by 5 minutes of sonication. All substrates were cleaned by sonicating in IPA for 15 minutes, followed by blow drying. For film deposition, 250 μL of the dispersion was spun dispensed clean fused silica substrate and spin-coated at 1000 rpm for 60 seconds. The back of the substrate was masked with tape to reduce unwanted accumulation along the edges and back surface. This masking step helped minimize edge defects that may otherwise propagate during PMMA coating. By limiting contamination and patchiness at the film edges, the wetting behavior of the PMMA solution was improved. Removal of the tape prior to PMMA deposition did not affect this improvement, as the initial nanoplatelet film morphology had already been enhanced. Films were dried in a fume hood under ambient conditions, then refrigerated for 1 hour. Samples were stored under vacuum at 30 °C overnight to ensure complete drying and allow for the repair of minor surface defects.

S1.1.5 PMMA Coating and Thermal Annealing

The following day, a PMMA overlayer was applied. PMMA A6 was diluted with toluene in a 1:1 volume ratio and spin-coated under two different conditions:

- Sample 1: 1250 rpm for 40 seconds, resulting in a film thickness of approximately 257 nm.
- Sample 2: 750 rpm for 40 seconds, resulting in a film thickness of approximately 295 nm.

After spin-coating, samples were thermally annealed at 100 °Cfor 5 minutes to promote film uniformity and improve adhesion.

S1.1.6 Design, Fabrication, and Characterization of Polaritons and Control Samples

Each polariton structure consisted of four layers: a distributed Bragg reflector (DBR), a $CsPbBr_3$ nanoplatelet layer, a polymer layer, and an aluminum mirror. Custom DBRs were obtained from Omega Optical LLC, USA. The thickness of $CsPbBr_3$ nanoplatelets film which make up the active layer was determined using transfer matrix simulations based on a reference film with known optical density. The samples used in polariton fabrication were prepared according to the method detailed above and redispersed in 1.5 mL of hexane. Spin parameters for the polymer layer were determined by first spin-coating PMMA solution on a clean silicon wafer, curing the film at 100 °C for 5 minutes, and measuring its thickness using ellipsometry. A 15 nm thick aluminum layer was deposited on top of the polymer layer by physical vapor deposition at a rate of 5 $\AA$/second. Control samples were prepared using the same method as the polariton samples, except that optical-grade fused silica substrates were used in place of DBRs.

S1.1.7 Optical Characterization

Nanoplatelet films with and without PMMA capping were prepared by spin-coating the solution on fused silica substrates as described above. Optical density was measured using a Jasco V-770 UV-Vis-NIR spectrophotometer. Transmission and reflection spectra were recorded using a custom-built spectrometer comprising a Thorlabs SLS204 deuterium light source fiber-coupled to an achromatic collimator, with detection by an OceanFX spectrometer. Angle-resolved measurements were performed using precision motorized rotation stages equipped with DC servo motor controllers for both the sample and detector. This setup allowed transmission and reflection spectra to be measured in the same reference plane. The dispersive and anti-crossing behavior of the polaritons was characterized by measuring reflection spectra at multiple incident angles, using a blank DBR as the background reference for each measurement.

Angle-resolved photoluminescence spectra were measured using an apparatus detailed in our previous study.[1] We excited the sample at 3.06 eV (405 nm) using a CW laser, controlled the angles of incidence and detection using a manual rotation stage, and collected the emitted light in a backscattering geometry using a parabolic mirror. Notch filters were placed in front of the slit of a spectrograph affixed with a CCD camera. Incident laser powers were maintained below 2 μW to avoid photo-damaging the sample. Measurements were made at several spots on the sample to ensure reproducibility.

S1.2 Computational Methods

We developed a method to estimate the optical properties of oriented thin films of $CsPbBr_3$ nanoplatelets based on the Kramers-Kronig relations. First, we took the transmission spectrum shown in the top left panel of Figure S4 to construct the imaginary part of the material's index of refraction. This construction allowed us to estimate the film thickness. Second, we used a numerical fixed-point KK transformation to calculate the real part of the material's index of refraction. Lastly, we used these optical properties to calculate the transmission and reflection spectra of the cavity samples we fabricated experimentally. Based on this methodology, we sought to form exciton cavity polaritons using n = 2 $CsPbBr_3$ nanoplatelet thin films with a PMMA spacer layer. To understand the behavior of these structures, we also formed control samples where $CsPbBr_3$ nanoplatelet thin films formed using the same synthetic steps are cast on optical grade fused silica substrates and capped with PMMA layers of the same thicknesses as those used to make exciton cavity polaritons. We compare the spatial structures of these designs in Figure S5.

S2 Comparison of Absorption of $CsPbBr_3$ Nanoplatelet Thin Films Formed under Different Processing Conditions

Figure S1 compares the absorption spectra of $CsPbBr_3$ thin films processes with different methods following their fabrication with the parameters described in section S1.1.4. The top right and

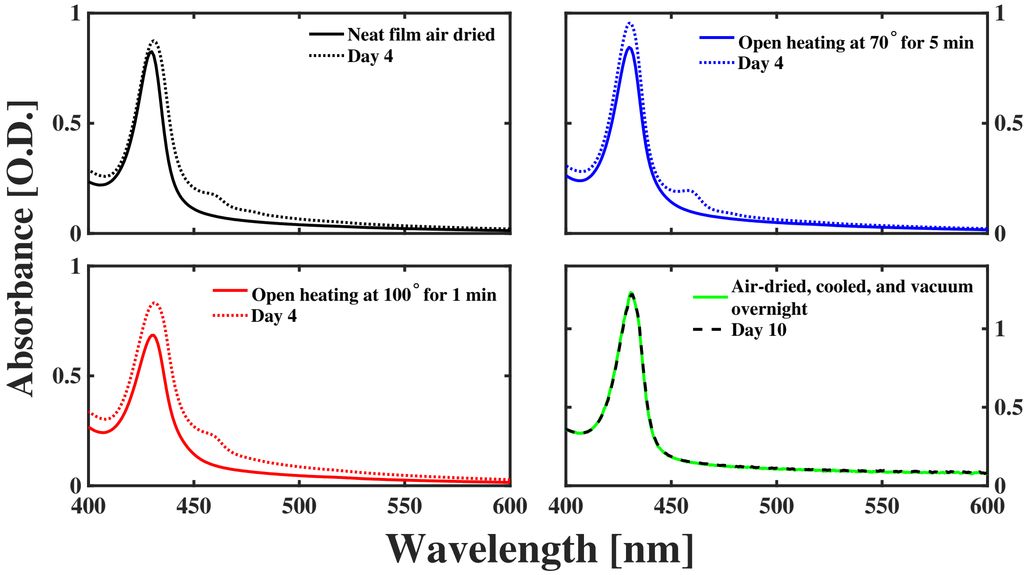

Figure S1: **Top left panel**: comparison between a thin film comprised of n = 2 $CsPbBr_3$ NPLs immediately following is fabrication to that of the same sample 4 days later, which shows an relatively increased baseline and a peak corresponding to the n = 3 $CsPbBr_3$ NPL species. **Top right panel**: comparison between a thin film comprised of n = 2 $CsPbBr_3$ NPLs following 5 minutes of heating at 70° after initial fabrication to that of the same sample 4 days later, which shows an relatively increased baseline and a peak corresponding to the n = 3 $CsPbBr_3$ NPL species. **Bottom left panel**: comparison between a thin film comprised of n = 2 $CsPbBr_3$ NPLs following 5 minutes of heating at 100° after initial fabrication to that of the same sample 4 days later, which shows an relatively increased baseline and a peak corresponding to the n = 3 $CsPbBr_3$ NPL species. **Bottom right panel**: comparison between a thin film comprised of n = 2 $CsPbBr_3$ NPLs following air drying, refrigeration, and vacuum drying for 12 hours after initial fabrication to that of the same sample 10 days later, which shows no signs of a peak corresponding to the n = 3 $CsPbBr_3$ NPL species.

bottom left panels of Figure S1 show similar results for two separate methods of stabilizing the structures of $CsPbBr_3$ nanoplatelet thin films following sample processing. We find the appearance of a prominent n = 3 excitonic peak in the sample absorption spectrum following sample heating to 70° and 100° for no more than 5 minutes. We stabilize the n = 2 only when we use all the processing steps detailed in the Methods section above.

Figure S2 compares how the application different processing methods affects the absorption spectra of $CsPbBr_3$ thin films following their being capped with polymethyl methacrylate (PMMA). These comparisons show that applying the full processing techniques discussed above can stabilize the NPL thin films sufficiently to maintain their absorption spectrum following encapsulation with

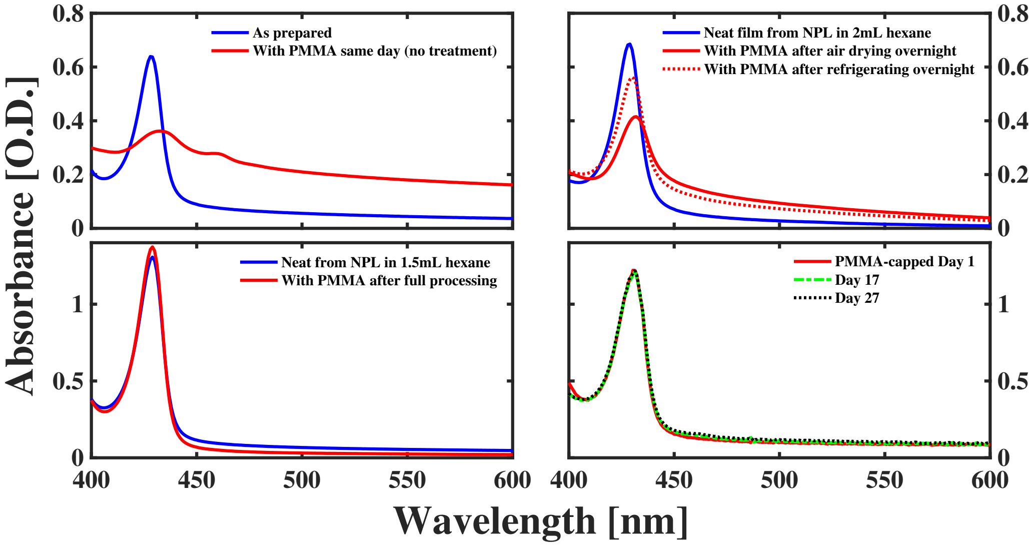

Figure S2: **Top left panel**: comparison between a thin film comprised of n = 2 $CsPbBr_3$ NPLs immediately following is fabrication to that of the same sample after encapsulation with a ∼250 nm PMMA layer, which shows an relatively increased baseline and a peak corresponding to the n = 3 $CsPbBr_3$ NPL species. **Top right panel**: comparison between a thin film comprised of n = 2 $CsPbBr_3$ NPLs immediately after initial fabrication to that of an air-dried sample and a refrigerated sample after encapsulation with ∼250 nm PMMA layers, which both show relatively increased baselines and shifts of the main excitonic absorption peak. **Bottom left panel**: comparison between a thin film comprised of n = 2 $CsPbBr_3$ NPLs following air drying, refrigeration, and vacuum drying for 12 hours after initial fabrication to that of the same sample after encapsulation with a ∼250 nm PMMA layer, which shows no signs of a peak corresponding to the n = 3 $CsPbBr_3$ NPL species. **Bottom right panel**: time evolution of the absorption spectrum of the sample shown in the bottom left panel at 1 day, 17 days, and 27 days after its initial fabrication, which shows no signs of additional peaks.

a PMMA layer.

S3 Effects of Capping Stable $CsPbBr_3$ Films with Aluminum

Figure S3 shows the transmission and reflection spectra of PMMA-capped $CsPbBr_3$ NPL thin films before and after deposition of a ∼15 nm layer of Al on the polymer surface. These comparisons indicate that we retain the pertinent features of the absorption spectra, which include the appearance of single peak in the transmission spectrum that reduces the transmitted intensity by 70% relative the baseline signal.

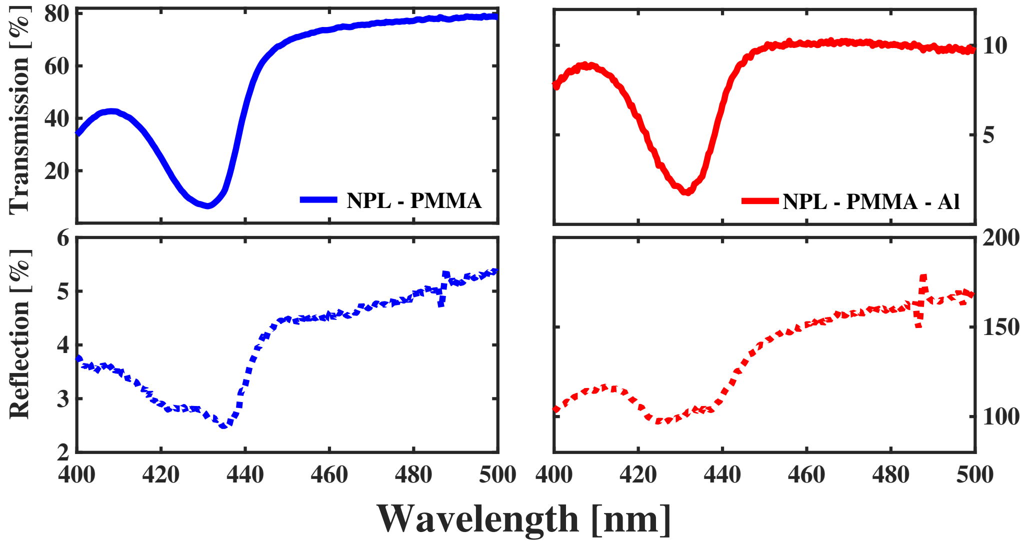

Figure S3: **Left panels**: transmission (top) and reflection (bottom) spectra of a stabilized n = 2 n = 2 $CsPbBr_3$ NPL thin film after being encapsulated with a ∼250 nm PMMA layer. **Right panels**: transmission (top) and reflection (bottom) spectra of a PMMA-capped, stabilized n = 2 n = 2 $CsPbBr_3$ NPL thin film after being encapsulated with a ∼15 nm Al layer.

S4 Structure of Fabry-Pérot Micro-resonators for Exciton Cavity Polariton Formation

Figure S4 compares the structural schematics of our precision designed Fabry-Pérot micro-resonator to that of control sample. As seen by inspection of Figure S4, the Fabry-Pérot micro-resonator contains a commercially fabricated distributed Bragg reflector (DBR), which is absent in the control. Besides this difference, all other attributes of the different types of samples are fabricated using the same parameters, which are detailed in the Methods section above.

S5 Comparison of Angle-Resolved Photoluminescence Peaks between Control and Cavity Samples

Figure S5 compares the dependence of the photoluminescence peak energy on the detection angle (θ_{det}) for a exciton cavity polariton sample to that of a control sample. This comparison shows that

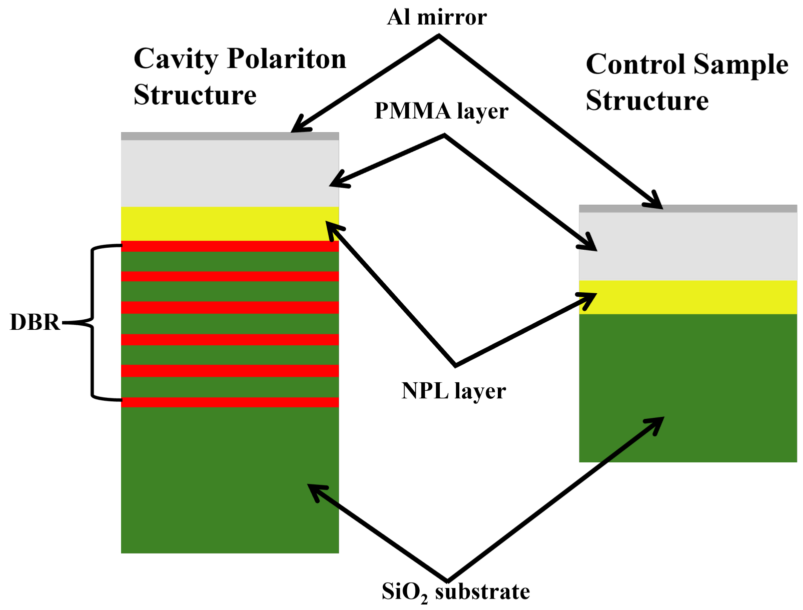

Figure S4: Schematic comparison between the structures Fabry-Pérot (left) and control (right) samples we used to assess the effects of strong light-matter coupling with $CsPbBr_3$ NPL films. Al mirror is a $\sim$15 nm layer of aluminum, PMMA layer is a 200-300 nm film of polymethyl methyacrylate, NPL layer is the $\sim$100 nm thin film of $CsPbBr_3$ nanoplatelets, DBR is a distributed Bragg reflection, and each sample is formed on an optical quality silicon dioxide (SiO_2) substrate.

while the energy of the peak in the control sample does not vary systematically as a function of θ_{det}, the position of the peak in the cavity sample disperses as a function angle. This angular dispersion matches what is expected from the lower polariton state of the hybrid light matter system, which further confirms our ability to reach the strong light-matter coupling limit using $CsPbBr_3$ stabilized with our processing approach.

S6 Comparison of Angle-Resolved Photoluminescence Intensity from Experiment and Model

Figure S6 compares the dependence of the photoluminescence peak intensity on the incidence (θ_{inc}) and detection (θ_{det}) angles for a cavity sample filled with n = 2 $CsPbBr_3$ NPLs to that of a

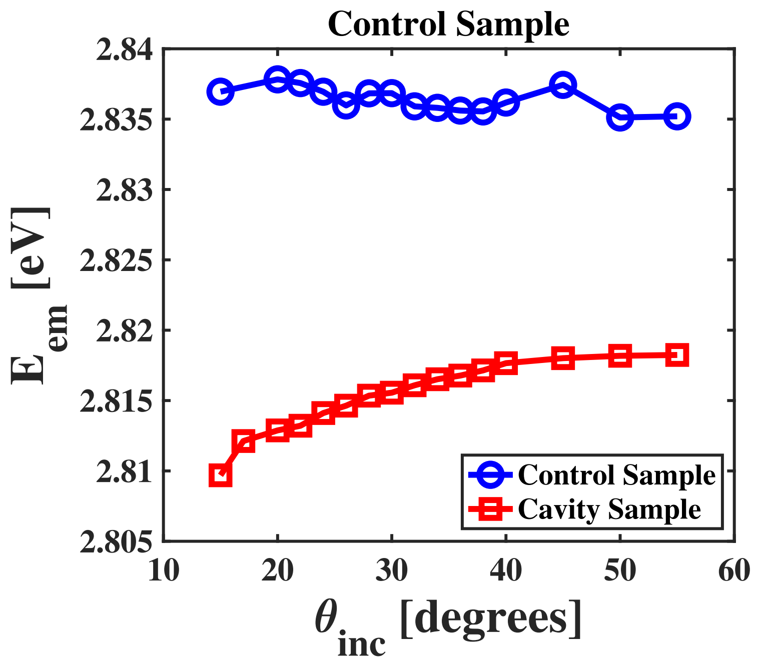

Figure S5: Comparison of the detection angle (θ_{det}) dependence of the 441 nm peak in the photoluminescence spectrum of a $CsPbBr_3$ NPL film embedded in a precision fabricated Fabry-Pérot micro-resonator (red squares) to that of a 438 nm peak in the control sample shown in Figure 4 (blue circles).

nonlinear regression fit of the experimental results to the following equation,

$$I(\theta_{inc}, \theta_{det}) = I_0 e^{-[(\theta_{inc}-\theta_0)/2\Delta\theta]^2} \cos^2\left[\pi \frac{d_{eff}\sin(\theta_{det})}{\lambda_{em}}\right]. \quad \text{(S1)}$$

This comparison shows that we can model the experimental data as a function of θ_{inc} and θ_{det} adequately using Eq. (S1) and we find estimated fit parameters close to those used to produce the model results in Figure 3(d) of the main manuscript. This finding indicates that the PL intensity becomes modulated due to interference between light paths that begin at different spatial positions within the cavity sample and helps supports our conclusion that we reach the strong light-matter coupling limit using the stabilized n = 2 $CsPbBr_3$ NPLs. Furthermore, the PL intensity becomes enhanced when the upper polariton state goes into resonance with the incident laser beam.

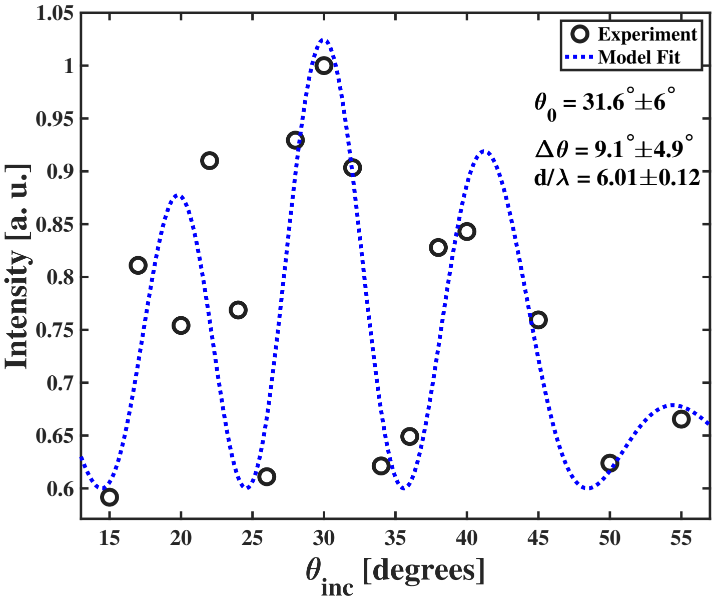

Figure S6: Comparison of the dependence of the 441 nm peak in the photoluminescence spectrum of a $CsPbBr_3$ NPL film embedded in a precision fabricated Fabry-Pérot micro-resonator on incidence (θ_{inc}) and detection (θ_{det}) angle dependence (black circles) to that of a nonlinear regression fit to Eqn. (S1) (dotted line.

References

(1) Odewale, E. O.; Wanasinghe, S. T.; Rury, A. S. Assessing the Determinants of Cavity Polariton Relaxation Using Angle-Resolved Photoluminescence Excitation Spectroscopy. *The Journal of Physical Chemistry Letters* **2024**, *15*, 5705–5713.
