# Supplementary material for: Stabilizing Ultrathin CsPbBr3 Nanoplatelet Films for Deterministic Strong Light-Matter Coupling
Source: J Phys Chem Lett. 2026 Feb 25;17(10):2877–85. doi: 10.1021/acs.jpclett.5c03989 (PMC12990107; doi:10.1021/acs.jpclett.5c03989)
Supplement: Supplementary file 2 [file jz5c03989_si_002.pdf]

jz-2025-039896.R1

Name: Peer Review Information for "Stabilizing Ultrathin CsPbBr₃ Nanoplatelet Films for Deterministic Strong Light-Matter Coupling"

First Round of Reviewer Comments

Reviewer: 1

Comments to the Author

The authors report a strategy to stabilize $CsPbBr_3$ nanoplatelets for cavity polariton applications through the design of a Fabry–Perot micro-resonator. The study provides interesting insights into strong light–matter coupling and related quantum phenomena. However, substantial revisions are required before the manuscript can be considered for publication.

Major comments:

1. The authors should include a schematic illustration clarifying the role of $CsPbBr_3$ nanoplatelets within the microcavity, as well as the underlying mechanism of strong light–matter coupling in the Fabry–Perot resonator.

2. The manuscript states that PMMA post-treatment improves the structural stability of $CsPbBr_3$ nanoplatelets. The physical or chemical origin of this stabilization should be discussed in more detail. In addition, the authors should comment on whether other polymers could provide similar stabilization effects.

3. The influence of the refractive index of PMMA on the strong light–matter coupling performance should be addressed. Would polymers with higher refractive indices be expected to further enhance or modify the coupling behavior?

4. The authors claim an edge-up orientation of $CsPbBr_3$ nanoplatelets in the thin film based on XRD results. Are there additional characterization techniques that could further support this structural assignment?

5. To better highlight the advantages of $CsPbBr_3$ nanoplatelets in microcavity applications, the authors should consider fabricating and characterizing microcavities based on $CsPbBr_3$ nanocubes as a reference or control sample.

Author's Response to Peer Review Comments:

Dear Editor,

Thank you for sharing your decision about our manuscript with us. We have revised the document extensively based on feedback from the reviewer. We sincerely thank the reviewer for their time and efforts in reviewing our manuscript and for the thoughtful, constructive feedback. We address each reviewer comment below. We denoted any text added to the manuscript in response to the reviewer comments in blue. We have also provided a markup version of the manuscript that includes the additional text in blue font and line numbers. Thank you for considering our manuscript further for publication in *The Journal of Physical Chemistry Letters.*

Sincerely,

Aaron S. Rury

Response to Reviewer:

The reviewer provided important points for us to consider in revising our manuscript.

The reviewer states,

The authors should include a schematic illustration clarifying the role of $CsPbBr_3$ nanoplatelets within the microcavity, as well as the underlying mechanism of strong light–matter coupling in the Fabry–Perot resonator.

Author Response: We thank the reviewer for this insightful feedback. To clarify the role of $CsPbBr_3$ nanoplatelets within the microcavity and to illustrate the mechanism of strong light-matter coupling, we have prepared a schematic presented in Figure 3 of the revised manuscript. On one side, a pictorial depiction shows 2-monolayer nanoplatelets embedded in a typical Fabry-Perot microcavity, illustrating how cavity photons are confined between two mirrors and interact with excitonic transitions, leading to the formation of polaritons. On the other side, a spatial representation of the microcavity layers is shown together with a simulation of the electric field propagation, providing an intuitive view of how the optical field is confined and interacts with the nanoplatelets. These illustrations allow the reader to visualize both the physical structure of the microcavity and the underlying mechanism of strong light-matter coupling.

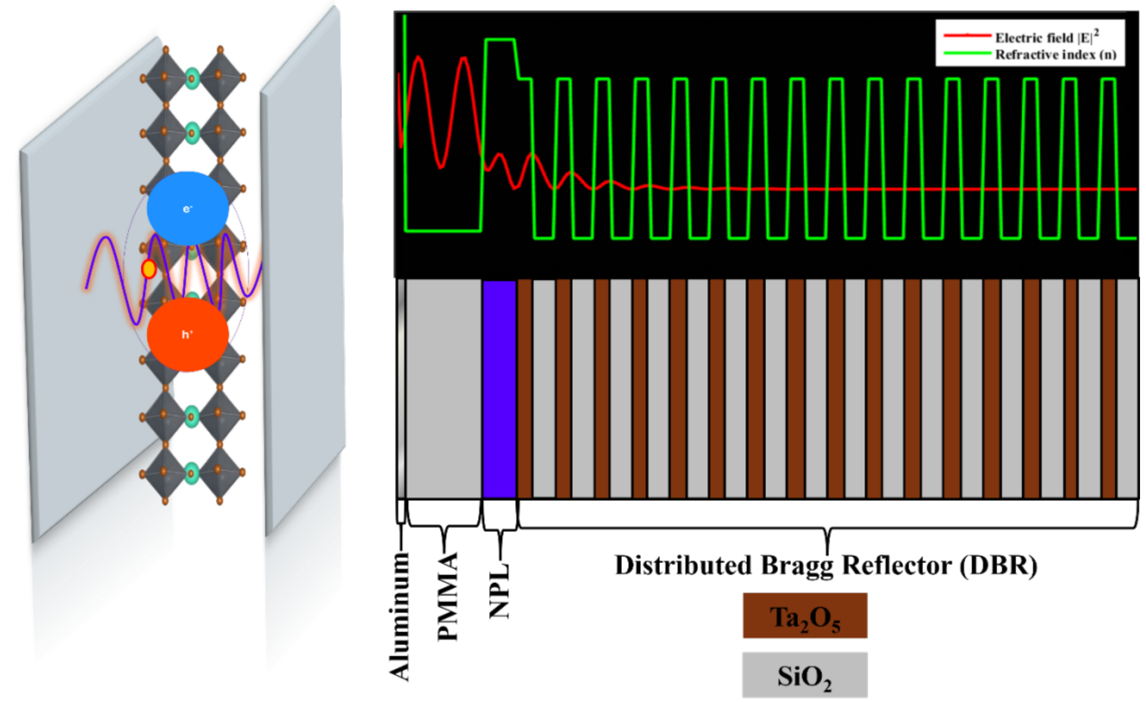

Figure 3: Left: Schematic of a 2-monolayer nanoplatelet embedded between cavity mirrors, showing an exciton (electron-hole pair) interacting with the oscillating cavity electric field. Top Right: simulated electric-field intensity (red) and refractive index profile (green) across the microcavity. Bottom Right: spatial representation of the cavity layers, including the DBR (alternating Ta_2O_5 and SiO_2), nanoplatelet layer (bluish purple), PMMA spacer, and aluminum mirror. The simulation highlights light confinement in the cavity and its overlap with the excitonic layer which results in strong light-matter coupling.

The corresponding discussion of Figure 3 has been added to page 4 of the manuscript.

Figure 3 illustrates the mechanism of strong light–matter coupling in a Fabry–Perot microcavity containing $CsPbBr_3$ nanoplatelets. On the left, 2-monolayer $CsPbBr_3$ nanoplatelets are sandwiched between two mirrors, with the exciton depicted as an electron-hole pair. The overlaid sinusoidal wave represents the oscillating cavity electric field, showing a likelihood of interaction between the exciton and the cavity photon. The right side of the schematic shows a spatial view of the cavity layers, highlighting the alternating high- and low-index regions of the distributed Bragg reflector, the active nanoplatelet layer, the polymer layer, and the aluminum mirror, representing a typical microcavity used in the study. We modeled the electric field propagation through the structure and combined the wave with a refractive index plot to illustrate how the electromagnetic wave travels across the different layers. The combination of the spatial schematic and the field simulation demonstrate how the confined optical field can overlap with excitonic transition to enable coherent energy exchange that drives strong light-matter interaction.

The reviewer states,

The manuscript states that PMMA post-treatment improves the structural stability of $CsPbBr_3$ nanoplatelets. The physical or chemical origin of this stabilization should be discussed in more detail. In addition, the authors should comment on whether other polymers could provide similar stabilization effects.

Author Response: We thank the reviewer for this comment. In the revised manuscript, we clarify that PMMA encapsulation is part of a broader sequence of post-synthetic treatments that enhances the stability of the films. The process we found most effective is air-drying the nanoplatelet films, refrigeration, and vacuum drying before PMMA deposition. Films treated this way were significantly more robust than those with direct PMMA deposition, as shown in Figure S1 (supplementary information). The PMMA layer primarily provides physical stability, protecting the nanoplatelets film from air and moisture, which are known to accelerate perovskite degradation. We also note that other polymers could provide similar stabilization if processed

carefully, with attention to solvent compatibility and annealing conditions to avoid film degradation. This discussion has been added to page 3 of the revised manuscript:

> *It is worth emphasizing that the primary improvement in stability arises from careful post-synthetic processing rather than from encapsulation alone. Steps such as air drying, refrigeration, and vacuum drying allow for subtle structural rearrangements within the perovskite lattice and enhance the optical density of the films, leading to improved stability of the ligand-passivated perovskite moieties. Following this pre-treatment, PMMA encapsulation provides an additional layer of protection, acting as a barrier against ambient moisture and oxygen. While the polymer layer alone cannot fully compensate for inadequate pre-processing, it helps preserve the structural and optical improvements achieved through postsynthetic treatment, enabling reliable integration of the nanoplatelets into microcavity devices.*
>
> *Other polymers could provide similar stabilization effects if processed carefully. For the $CsPbBr_3$ nanoplatelets in particular, the solvent used to dissolve the polymer must be compatible with the nanoplatelet film, meaning it should not dissolve or otherwise damage the perovskite layer formed from its original dispersion solvent. Optimizing deposition method and thermal treatment are also important to avoid film degradation. Overall, the combination of post-synthetic processing to stabilize the perovskite moieties, followed by polymer encapsulation for film protection, represents a practical and versatile method to maintain the properties of $CsPbBr_3$ nanoplatelets for optoelectronic and polaritonic applications.*

The reviewer states,

The influence of the refractive index of PMMA on the strong light–matter coupling performance should be addressed. Would polymers with higher refractive indices be expected to further enhance or modify the coupling behavior?

<u>Author Response:</u> We thank the reviewer for this important comment. To address it thoroughly, we simulated a microcavity structure using different refractive indices for the polymer layer. In these simulations, the polymer thickness was adjusted to maintain the cavity resonance condition when changing the refractive index, allowing us to focus primarily on the influence of the refractive index. With this constraint, our modeling shows that a higher-index polymer significantly modifies the cavity photon mode, thereby affecting the dispersion of the resulting polariton branches and causing a slight redshift of the polariton energy at normal incidence. In particular, both the lower and upper polariton branches become less dispersive, with a noticeable flattening of the lower polariton branch. This behavior is further accompanied by a reduction in the intensity of the reflection spectra, which we attribute in part to the reduced refractive index mismatch at the polymer-nanoplatelet interface, leading to lower Fresnel reflection and a modified cavity field distribution through the nanoplatelet layer. These results demonstrate that the polymer refractive index provides an effective handle for tailoring polariton dispersion and mode characteristics under strong light-matter coupling conditions. To highlight this point, we have added the following text to page 4 of the revised manuscript,

> *The polymer layer influences the microcavity primarily through its refractive index, while the polymer thickness is adjusted to satisfy the cavity resonance condition. Increasing the polymer refractive index modifies the cavity photon dispersion, leading to flatter polariton branches, particularly for the lower polariton, along with a reduction in polariton branch intensity and a small redshift of the polariton energy at normal incidence. Furthermore, a higher refractive index reduces the refractive index mismatch at the polymer-nanoplatelet interface, thereby decreasing Fresnel reflection and slightly altering the cavity field distribution within the nanoplatelet layer. These effects may occur without a significant change in the Rabi splitting if the exciton density determined by the thickness of the absorber remains the same.*

The reviewer states,

The authors claim an edge-up orientation of $CsPbBr_3$ nanoplatelets in the thin film based on XRD results. Are there additional characterization techniques that could further support this structural assignment?

Author Response: We thank the reviewer for raising this point. We realize that our original wording may have caused some confusion. To clarify, our $CsPbBr_3$ nanoplatelet films are predominantly face-down, and we did not specifically set out to characterize edge-up nanoplatelets in this study. Our reference to the minor peaks generally ascribed to edge-up oriented NPLs was intended to highlight the increase in homogeneity observed after our post-processing sequence. We have now clarified this explicitly in the revised manuscript, making the connection between XRD changes, film homogeneity, and enhanced light–matter coupling clear by replacing the text highlighted in red with the updated blue text on page 3 of the revised manuscript:

> ~~*Additionally, we find smaller contributions from the so-called edge-up orientation of the underlying NPLs, which correspond to particles whose long axis lies perpendicular to the substrate surface. These features appear with significantly smaller intensities at 2θ values of 15.61° and 31.01°.*~~
>
> *Additionally, we find minor peaks at 2θ values of 15.61° and 31.01°, which are reported in the literature as belonging to the edge-up orientation of $CsPbBr_3$ NPLs, whose long axis lies perpendicular to the substrate surface. Despite their low intensities, the presence of these peaks suggests that a small fraction of nanoplatelets in our as-prepared samples adopt this orientation, implying some heterogeneity in the material.*
>
> ~~*Furthermore, the reduction in the intensity of the peaks corresponding to NPLs in the edge-up orientation suggests that we suppress the formation of these species within the film, which also helps to enable strong light-matter coupling as we discuss below. We propose that the post-processing methods we describe here help to stabilize the sub-lattice of organic ligands that cap the individual NPLs during the solution phase synthesis. This stabilization of the organic sub-lattice allows the CsPbBr3 quantum wells to align more completely (increased XRD intensity), relax structurally (shift to higher 2θ values), pack into tighter spatial*~~

~~*configurations (increased spacing between the Bragg peaks), and relax into uniform orientations relative to the substrate surface (reduced contribution from edge-up configuration).*~~

Based on these changes observed in XRD measurements, we propose that the post-processing methods we employed help stabilize the sub-lattice of organic ligands that cap the individual NPLs during the solution-phase synthesis. These changes indicate improved crystallinity and stabilization of the organic sub-lattice, which allows tighter packing of the $CsPbBr_3$ NPLs within the superlattice. Notably, the intensity of the minor peaks generally ascribed to edge-up oriented NPLs decreases, indicating that the post-processing further enhances the homogeneity of the film. This more uniform, face-down alignment facilitates stronger coupling to cavity photons in our spectroscopic measurements. Overall, the observed XRD changes confirm that the post-processing steps help align, structurally relax, and stabilize the NPLs in their dominant face-down orientation, thereby facilitating strong light–matter coupling.

The reviewer states,

To better highlight the advantages of $CsPbBr_3$ nanoplatelets in microcavity applications, the authors should consider fabricating and characterizing microcavities based on $CsPbBr_3$ nanocubes as a reference or control sample.

<u>Author Response:</u> We thank the reviewer for this valuable suggestion. We agree that nanocubes could serve as a useful reference system, but fabricating such microcavities would require substantial additional work, involving redesigning the cavity to match the nanocube absorption energy, engaging external vendors to fabricate distributed Bragg reflectors with appropriate stopband in the region of interest, synthesizing the materials and optimizing deposition protocols, before going on to perform full optical characterization, including angle-resolved reflection and photoluminescence measurements. Implementing this comparison would therefore require significant additional efforts that are beyond the scope of the present revision. $CsPbBr_3$ nanoplatelets are interesting for exploring cavity polaritons because they combine large excitonic

oscillator strength, narrow excitonic linewidths, and in-plane dipole orientation, all of which favor strong light–matter coupling. Despite these advantages, they are more difficult to precisely fabricate and integrate into microcavities than nanocubes. The objective of this manuscript is to show how these challenges can be overcome and to characterize the resulting nanoplatelet-based microcavity polaritons. To better highlight the advantages of $CsPbBr_3$ nanoplatelets in microcavity applications as advised, we included the following text on page 5 in the revised manuscript:

> *Despite how readily the nanocubes form and their perceived stability compared to the nanoplatelets, the nanoplatelets offer distinct advantages as the active layer for polariton formation in microcavities. Compared to nanocubes, the platelets combine large excitonic oscillator strength, narrow excitonic linewidths, tunable bandgap, and planar dipole orientation, making them particularly well suited for exploring strong light–matter coupling in a Fabry-Perot microcavity. One key advantage is the ability to tune the excitonic bandgap by controlling the number of monolayers, allowing precise alignment with the cavity photon without altering the chemical composition of the perovskite lattice.*
>
> *In this study, the thickness of $CsPbBr_3$ nanoplatelets was carefully controlled during synthesis to achieve the desired monolayer number, enabling fine tuning of the exciton energy across the visible spectrum. By contrast, nanocube bandgaps are primarily tuned via chemical substitution, providing less continuous control and altering the lattice structure. Also, the quasi-2D geometry of nanoplatelets leads to stronger quantum confinement and higher exciton binding energies than nanocubes, stabilizing the nanoplatelets' excitons and enhancing their interaction with cavity photons. The nanoplatelets also exhibit longer exciton coherence lengths and narrower homogeneous linewidths, which improve the visibility of polariton peaks.*
>
> *In addition, the planar morphology and anisotropic shape allow preferential in-plane orientation during film deposition, maximizing overlap with the cavity electric field, whereas nanocubes tend to adopt random orientations that reduce effective coupling. Together with the higher effective density of aligned dipoles in the film, these features increase the collective light–matter interaction strength and*

Rabi splitting. Studies of polariton formation in other colloidal nanoplatelet systems, such as CdSe, further confirm the efficiency of nanoplatelet exciton states in hybridizing with cavity photons. Overall, these structural and optical properties make $CsPbBr_3$ nanoplatelets especially advantageous for microcavity polariton studies and the development of tunable strong-coupling devices, despite the difficulty around stabilizing and incorporating them into microcavities.
